# Frequency and Duration of Advertising on Popular Child-Directed Channels on a Video-Sharing Platform

**DOI:** 10.1001/jamanetworkopen.2021.9890

**Published:** 2021-05-13

**Authors:** Samantha L. Yeo, Alexandria Schaller, Michael B. Robb, Jenny S. Radesky

**Affiliations:** 1 Department of Pediatrics, University of Michigan Medical School, Ann Arbor; 2 Common Sense Media, San Francisco, California

## Abstract

This cross-sectional study quantifies the number and duration of distinct advertisements that appear during child-directed videos on a video-sharing platform as well as the frequency of age-inappropriate ads.

## Introduction

Free video platforms are popular among young children, but their advertising content and duration are not regulated by federal policy. A recent analysis of online videos viewed by young children showed that 85% contained ads, of which 20% had age-inappropriate content, such as violence or political messages.^1^ Television advertising is regulated because of children’s limited abilities to recognize and resist ad persuasion.^2^ No prior research, to our knowledge, has quantified the number or duration of distinct ads that appear during child-directed YouTube videos, which this study aimed to examine, along with the frequency of age-inappropriate ads.

## Methods

Adapting methods from Alruwaily et al^3^ for this cross-sectional study, we identified the top 10 most-subscribed English-language made-for-kids channels on YouTube.com^4^ as of December 16, 2020. Because this was a content analysis of publicly available data, this study did not require institutional review board approval, per the policy of the University of Michigan. For each channel, we watched the 5 videos with the highest view counts, confirming that the videos were child-directed based on the video’s availability on the video-sharing platform’s counterpart for children younger than 13 years and the lack of a comments section. We chose to view only 5 videos per channel based on observations that ads typically appear in similar patterns within channels. One investigator (S.L.Y.) watched each video once in its entirety, recorded metadata (ie, video length, view count); ad number, duration, and placement (ie, preroll, interstitial, postvideo, banner, and sidebar); and coded ad age-appropriateness (reliability κ = 0.951).^1^ The Table contains the coding scheme.

**Table.** Advertising Frequency and Duration in the 10 Most Popular Child-Directed Channels on the Video-Sharing Platform

Channel name	Subscriber count, millions^a^^,^^b^	Video view count, mean (SD), No.^a^	Ads per video, mean (SD), No.						Ad duration per min of video, mean (range), min^c^		Total ad duration, without skips/with skips, s	Age-inappropriate ads, No./total No. (%)^d^
			Total	Preroll	Interstitial	Postvideo	Banner	Sidebar	Without skips	With skips		
Cocomelon—Nursery Rhymes	100	2 430 314 350 (638 060 397)	3.2 (0.8)	1.4 (0.6)	0	0.4 (0.6)	0	1.4 (0.6)	0.35 (0.09-1.00)	0.05 (0.03-0.07)	294.0/45.0	0/16 (0.0)
Kids Diana Show	70.9	830 806 719 (222 916 802)	3.8 (0.8)	1.4 (0.9)	0	0.8 (1.1)	0.4 (0.6)	1.2 (0.5)	0.34 (0.09-0.75)	0.03 (0.02-0.04)	755.0/50.0	0/19 (0.0)
Like Nastya	65.6	604 933 429 (152 060 961)	5.6 (1.7)	1.2 (1.1)	0.4 (0.9)	1.6 (0.9)	0.8 (0.8)	1.6 (0.6)	1.61 (0.90-2.30)	0.05 (0.03-0.06)	3127.0/100.0	3/28 (10.7)
Vlad and Niki	59.7	518 613 694 (62 429 997)	3.2 (0.5)	0.8 (1.1)	0	0.8 (0.8)	0.6 (0.6)	1.0 (0)	0.81 (0.46-1.42)	0.06 (0.05-0.08)	739.0/55.0	2/16 (12.5)
ChuChu TV Nursery Rhymes and Kids Songs	43.1	1 728 261 952 (945 373 493)	6.0 (4.7)	1.2 (1.1)	1.2 (2.7)	0.2 (0.5)	0.8 (0.5)	2.6 (1.5)	0.38 (0.09-0.67)	0.02 (0.01-0.04)	961.0/85.0	3/30 (10.0)
Pinkfong! Kids’ Songs & Stories	42.0	1 825 969 409 (3 161 385 589)	3.0 (0)	0.8 (0.5)	0	0.2 (0.5)	0.2 (0.5)	1.8 (0.5)	0.25 (0.04-0.87)	0.05 (0.04-0.07)	168.0/30.0	4/15 (26.7)
LooLoo Kids—Nursery Rhymes and Children’s Songs	37.5	1 494 319 680 (1 638 834 674)	7.2 (7.7)	0.2 (0.5)	1.2 (2.7)	1.0 (0.7)	1.4 (1.5)	3.4 (3.1)	0.70 (0.21-1.32)	0.06 (0.02-0.10)	1085.0/95.0	4/36 (11.1)
Little Baby Bum—Nursery Rhymes & Kids Songs	34.3	1 225 918 605 (645 940 991)	14.0 (16.6)	0.8 (0.8)	4.6 (6.4)	0	3.0 (3.7)	5.6 (6.2)	0.29 (0.04-0.66)	0.03 (0.02-0.04)	2731.0/210.0	2/70 (2.9)
Toys and Colors	27.6	386 450 326 (85 333 625)	5.2 (2.4)	1.0 (1.0)	0.4 (0.9)	0.6 (0.9)	0.6 (0.9)	2.6 (0.9)	0.41 (0.13-0.74)	0.04 (0.01-0.05)	725.0/65.0	0/26 (0.0)
Ryan’s World	27.5	825 505 656 (741 271 361)	6.0 (1.6)	0.8 (1.1)	0.4 (0.9)	1.6 (0.9)	0.6 (0.6)	2.6 (1.1)	0.47 (0.26-0.85)	0.04 (0.02-0.06)	954.0/85.0	0/30 (0.0)
Total, mean (SD)	NA	NA	5.7 (3.3)	1.0 (0.4)	0.8 (1.4)	0.7 (0.6)	0.8 (0.9)	2.4 (1.4)	0.56 (0.04-2.30)	0.04 (0.01-0.10)	11 539.0/820.0	18/286 (6.3)

^a^ As of December 16, 2020.

^b^ Subscriber counts are reported to 3 significant figures, which is what is available on the respective channel pages on the video-sharing platform.

^c^ Excludes sidebar ads.

^d^ Reliability for age-inappropriateness was calculated for 2 coders simultaneously watching 50 videos from these child-directed channels, using the following scheme: 1, appropriate for child younger than 13 years (eg, education games, learning websites, toys for kids); 2, neutral (content is not harmful for child viewing but is not specifically targeted toward the age group, eg, colleges, home appliances); and 3, age-inappropriate for child younger than 13 years or targeted toward adults (eg, violent games, dating websites, politics).

We calculated the duration of ads by summing the duration of preroll, interstitial, postvideo, and banner ads (ie, ads that disrupt viewing; sidebar ads last the entire video and do not obscure it). We created 2 ad duration variables: total duration if not skipped and total duration if skipped after 5 seconds (if skip option provided), adjusting both for video duration. For each video and channel, we calculated the proportion of age-inappropriate ads. Descriptive summary statistics were calculated in Excel 2016 (Microsoft).

## Results

The 50 videos sampled contained a total of 286 ads, with a mean of 5.72 ads per video (SD, 3.25; range 2.00-38.00). Sidebar ads were most common (119 [41.6%]), followed by preroll (48 [16.8%]), banner (42 [14.7%]), interstitial (41 [14.3%]), and postvideo (36 [12.6%]) ads. Overall, the mean total duration of ads (not skipped) was 0.56 minutes per minute of video (median, 0.42 minutes per minute of video; range, 0.04-2.30 minutes per minute of video) and 0.04 minutes per minute of video if skipped (median 0.04 minutes per minute of video; range, 0.01-0.10 minutes per minute of video) (Table). Age-inappropriate ads appeared 18 times (6.3%) in 6 of 10 channels (60.0%) and usually comprised banner or sidebar ads for violent video games.

There was variability in ad frequency and duration between channels (Table). For example, Little Baby Bum had a mean of 14 ads per video and had the most interstitial ads, while Pinkfong! had a mean of 3 ads per video. If not skipped, ads on the Like Nastya channel were longer than the videos (mean, 1.60 ad minutes per minute of video; range, 0.90-2.30 ad minutes per minute of video); unskipped advertisement duration ranged from 11% to 80% of video duration on other channels.

## Discussion

This analysis of advertising frequency and duration on the most popular child-directed online video channels found that advertising load and age-appropriateness varied widely. On some channels, unskipped ads played for as much time, or longer than, the video itself and exceeded television ad limits.^5^ Although research suggests that young children can identify preroll ads on video-sharing platforms,^6^ it is not known how frequently they skip ads in practice. In future policy discussions, attention should be paid to the dose of advertising children receive—and the resulting profits channels receive—during video viewing. Video-sharing platforms could consider placing restrictions on advertising load and using human review for age-appropriateness of ads. Clinicians can guide parents on finding ad-free video platforms or helping children identify and skip ads.^2^ Limitations of this study include its focus on English-language videos and the dynamic nature of video popularity.
